# Mesostigmata diversity by manure type: a reference study and new datasets from southwestern Iran

**DOI:** 10.1007/s10493-022-00710-1

**Published:** 2022-03-31

**Authors:** Sara Farahi, Parviz Shishehbor, Alireza Nemati, M. Alejandra Perotti

**Affiliations:** 1 Department of Plant Protection, Faculty of Agriculture, Shahid Chamran University of Ahvaz, Ahvaz, Iran; 2 Department of Plant Protection, Faculty of Agriculture, Shahrekord University, Shahrekord, Iran; 3 Ecology and Evolutionary Biology Section, School of Biological Sciences, University of Reading, Reading, UK

**Keywords:** Gamasida, Species richness, Rarefaction, Dung, Biocontrol, Ahvaz, Iran, *Musca*, Muscidae

## Abstract

Manure-inhabiting Mesostigmata mites are important biological control agents of pest flies. However, the biodiversity of this mite community is mainly known from Europe and America, and especially from cattle manure. This study examined the diversity and abundance of Mesostigmata mites associated with various types of manure in an (intensive) agricultural region of the Middle East, i.e., the city Ahvaz and its suburbs, in southwest Iran. Mite samples were extracted from manure of cattle, buffalo, sheep, horse, poultry and quail in 30 livestock and poultry farms. In total, 40 species belonging to 24 genera and 16 families were identified. The most diverse families were Laelapidae with eight species, Macrochelidae with seven and Parasitidae with six. *Macrocheles muscaedomesticae* and *Uroobovella marginata* were the most widespread species, recorded in 28 and 27 out of 30 collection sites, respectively. Two species, *M. sumbaensis* and *U. marginata*, were found in all studied manures. Simpson’s diversity index recorded the highest diversity in buffalo and sheep manure. Real and theoretical species richness (rarefaction curves) were congruent in number of individuals. The presence of seven species of Macrochelidae in the manure confirms that these are important predators of the house fly for the region of Ahvaz and its suburbs. Members of the Parasitidae were highly prevalent, with one species known as a specialized predator of house fly eggs. This work aims to encourage further studies on the diversity of Mesostigmata in these agricultural settings, and further continue assessing the feasibility of these mites as effective biocontrol agents of filth flies in different types of manure and from different corners of the world.

**Supplementary Information:**

The online version contains supplementary material available at 10.1007/s10493-022-00710-1.

## Introduction

The Mesostigmata is a large, cosmopolitan taxon of parasitiform mites that comprises an extremely diverse variety of lifestyles and habitats (Lindquist et al. 2009). The greater number of species are free-living predators (Karg 1993), whilst many others are parasites or symbionts of mammals, birds, reptiles or arthropods and a few species feed on fungi, pollen, or even nectar (Walter and Proctor 1999). Free-living mesostigmatid mites are found in association with soil, litter, rotting wood, tree canopies, compost, carrion, animal manure and animal nests (Krantz 2009a; Lindquist et al. 2009). Of these varied habitats, soil – especially agricultural soils – have been so far the most investigated for mesostigmatids, although their diversity in other substrates from agricultural systems, such as manure-inhabiting mites, remains largely overlooked, despite their potential to control filth flies originated in these anthropogenic byproducts of agriculture.

The manure or dung of domestic animals including poultry are suitable media for the breeding of dipteran species from Muscidae and Fanniidae, such as *Musca domestica* L. or *Fannia canicularis* (L.) (Axtell 1964; Bohart and Gressitt 1951; Grisales and de Carvalho 2016; Hewitt 1912; Ito 1970; Kristofik 1984, 1988; Legner and Bowen 1973; Mihályi 1965; Nickolls and Disney 2001; Nuorteva 1963; Perotti 1998b, 2000a; Perotti and Bachmann 1999; Perotti and Lysyk 2003; Perotti et al. 2001; Perotti and Sardella 1998). Coprophilous Mesostigmata, mainly members of the Macrochelidae, Parasitidae, Laelapidae, Eviphididae, Pachylaelapidae and Uropodidae families are predator mites that usually feed on the eggs and larvae of Diptera, particularly muscid flies, and other micro-invertebrates from dung (Ciccolani 1979; Geden et al. 1988; Ho 1990; Perotti 1998a, 2001; Perotti et al. 2000; Rodriguez and Wade 1961; Rudzíska 1998; Schelvis 1991, 1994; Takaku et al. 1994; Wade and Rodriguez 1961). As a result, they are recognised biocontrol agents of filth flies in these habitats (Axtell 1963b, 1968, 1970a, 1970b; Azevedo et al. 2018; Ciccolani 1979; de Azevedo et al. 2015; De Jesus and Rueda 1992; Krantz 1983; Manning and Halliday 1994; Moya Borja 1981; Perotti 1996, 1998a, 1999a, 1999b, 2000b, 2001; Perotti and Brasesco 1996; Perotti et al. 2000; Rodrigueiro and Prado 2004; Rodriguez et al. 1970; Rodriguez and Wade 1961; Schelvis 1994; Wade and Rodriguez 1961). These same fly families, predated in their immature stage by mites, transport as phoretic carriers in their adult state the mites, that then found new predator-mite populations (Acs et al. 2017; Athias-Binche 1984, 1994; Axtell 1964; Bajerlein and Bloszyk 2003, 2004; Beresford and Suctcliffe 2009; Bloszyk et al. 2006; Fain 1998; Fain and Greenwood 1991; Farish and Axtell 1971; Glida et al. 2003; Greenberg and Carpenter 1960; Haloti et al. 2005; Krantz 2009b; McGarry et al. 1992; Niogret et al. 2010; Pereira and Castro 1947; Perotti 1998a; Perotti and Braig 2009; Perotti et al. 2010; Perotti and Brasesco 1996; Rodrigueiro and Prado 2004; Rudzíska 1998; Sato et al. 2018).

Studies on the potential of these mites as biocontrol agents started in the mid 1900’s with the work of Leitner (Germany), who carried out a comprehensive survey on the diversity of manure mites and their ecological roles, recording 121 species of 31 mite families (Leitner 1946a, 1946b). It was Filipponi (1955) (Italy) who first studied in detail the biology and ecology of the members of the family Macrochelidae as manure-inhabiting mites and determined the feasibility of mass production of macrochelids (Filipponi 1964). Later, Axtell (USA) conducted a series of experiments in poultry manure and found that Uropodidae was the most abundant, followed by Macrochelidae and then Parasitidae (Axtell 1961, 1963a, 1963b). He further investigated the control of house flies in poultry manure using *Macrocheles muscaedomesticae* (Axtell 1964, 1968, 1970a, 1970b). Moreover, Ito (1970) recorded Macrochelidae and Uropodidae as abundant mite families in livestock dungs in Japan. Krantz (1983) reported that 26 genera of mites representing nine families in the Mesostigmata order are known to be associated with dung beetles, and Halliday (2000) studied the Australian fauna of the mite genus *Macrocheles* (Macrochelidae) listing 49 species, most associated to ephemerous habitats such as manure. Only recently, new studies were carried out in other regions and continents. Özbek and collaborators studied the fauna of Macrochelidae in northern Turkey with the description of new species (Özbek 2017; Özbek and Bal 2013; Özbek et al. 2013, 2015a, 2015b; Özbek and Halliday 2015). Newer additions also include other disparate regions, for example South America, such as Azevedo et al. (2017) from Brazil, who described a new species of *Macrocheles*, and Porta et al. (2020), who reported *M. subbadius* from Argentina, although it was found on cattle dung pads in the Pampas region in 1994 (Perotti 1998a).

For the Middle East, particularly Iran, Zakeri et al. (2012) collected eight coprophilous species of Macrochelidae from the North (Golestan Province). Sobhani et al. (2017) recorded 11 species of Macrochelidae from manure in southern Iran (Fars Province). While Babaeian (2011) studied the fauna of Macrochelidae and Laelapidae in central Iran (Chaharmahal-va-Bakhtiari Province) and reported eight Macrochelidae species as well as seven Laelapidae species from manure. Kamali et al. (2001) provided a catalogue of mites and ticks (Acari) of Iran, listing 16 species of Mesostigmata which had been collected from manure. Ahangaran et al. (2012) surveyed the fauna of the edaphic and dung dweller mites of the superfamily Eviphidoidea (Acari: Mesostigmata) in northern Iran (western Mazandaran Province) and collected 14 species from manure. In terms of mite abundance it was found higher in Macrochelidae, followed by Pachylaelapidae and then Parholaspididae. Arjomandi et al. (2013) studied the fauna and diversity of the manure-inhabiting Mesostigmata in southeastern Iran (Kerman Province). They recorded 36 species in 14 families from a variety of manure, resulting in 31 species from cattle, which was more diverse than poultry and sheep manure which carried 14 and 13 species, respectively. Kazemi and Rajaei (2013) also studied cattle, sheep, chicken, poultry and camel manure and listed 72 species of manure-inhabiting Mesostigmata from Mazandaran, Guilan, Golestan, North Khorasan, Semnan, Tehran and Fars Provinces of Iran. Nemati et al. (2018) added 50 more manure species in their updated catalogue of the Iranian Mesostigmata (Acari). The most recent studies come from southwestern Iran (Farahi et al. 2020, 2019) including the new findings of this work.

Despite the importance of manure-inhabiting mesostigmatid mites as reliable biocontrol agents of dipteran pests, the topic remains understudied, and little is known of the biodiversity, and the biology of many of these species in the Middle East. The main objective of this work was to review and revive the interest for this topic, with the initial aim of studying the Mesostigmata diversity associated with six frequently generated manure types, as the most common farming product in the agricultural lands of southwest Iran.

## Materials and methods

The study was conducted using manure from 30 livestock and poultry farms of Ahvaz and its suburbs, Khuzestan Province, southwest Iran. The area is a semi-desert lowland part of the province, which is excessively warm and dry in the summer and the annual average rainfall does not exceed 230 mm.

### Field sampling and material preparation

Sampling was randomly done from the outermost layer of the manure (maximum depth of 20 cm) of five locations in each site, using a small trowel (Southwood and Henderson 2000). As not all types of manure were available in each site simultaneusly, the number of samples collected from different types of manure was deemed to be different in each case. Using Tullgren-Berlese funnels for up to 48 h (Tullgren 1918), mites were extracted from a total of 100 samples of six domestic animal manure types: cattle (33 samples), sheep (20), poultry (10), buffalo (27), horse (6) and quail (4), during 2015–2017. Quail manure was rarely found, which explains that only four quail manure samples were collected. The number of hours of funnel use depended on the relative humidity at the moment of extraction, if the humidity fell rapidly we used the funnels shorter, between 24 and 48 h.

The extracted specimens were preserved in 75% ethanol and placed in Nesbitt’s solution and lactophenol for clearing and then mounted in Hoyer’s medium on permanent microslides. The specimens were mounted under a stereo microscope (Olympus SZX12) and identifications were carried out with a Phase Contrast Olympus BX51 using up to 1000x magnification.

For analysis purposes, the data of the five locations in each site (by manure type) were pooled. Mesostigmata mites were identified, if possible, to the species level. Identification of species was done by the first and third authors (acarologists) using available literature including the original descriptions of the species (Berlese 1887, 1904a, 1904b, 1906, 1918; Bregetova et al. 1973; Costa 1966, 1967, 1968; Evans and Browning 1956; Evans and Hyatt 1963; Evans et al. 1961; Evans and Till 1979; Farrier and Hennessey 1993; Filipponi and Pegazzano 1962, 1963; Furman 1972; Halliday 2000; Hirschmann and Wiśniewski 1982; Hirschmann et al. 1991; Hughes 1976; Hyatt 1980; Hyatt and Emberson 1988; Karg 1962, 1989; Kazemi et al. 2014; Krantz 1962; Ma and Wang 1996; Niogret et al. 2007; Plumari and Kazemi 2012; Samšiňák 1964; Skorupski and Witaliński 1997; Witaliński 2017; Yao et al. 2019). The collected specimens (adults and identifiable nymphs, of which mainly deutonymphs were collected) were deposited in the Insect and Mite Collection of Ahvaz (IMCA), Department of plant protection, Shahid Chamran University of Ahvaz, except *Parasitus* sp. and some type material of *Trachygamasus karuni* which were deposited earlier in the Zoological Museum of the Jagiellonian University, Poland (Farahi et al. 2019). Two specimens belonging to Ascidae and Rhodacaridae were damaged and could not be identified.

### Data analyses

The number and relative frequency of each species were recorded for each manure type. Boxplots were generated with STATA (2014). The Mesostigmata species richness (using rarefaction), diversity (applying Simpson’s index) and Simpson’s measure of evenness of the studied manure mites were calculated for all samples, utilizing the software Ecological Methodology v.7.2 (Krebs 2011). Simpson’s index was compared between manure types, using a randomisation test with 10,000 re-samples in the SDR software v.4.1.2 (Seaby and Henderson 2006). The taxonomic distinctness index (Δ*) of Clarke and Warwick (1998) was also calculated by PAST v.4.06 (Hammer et al. 2001).

## Results

**Fig. 1:**
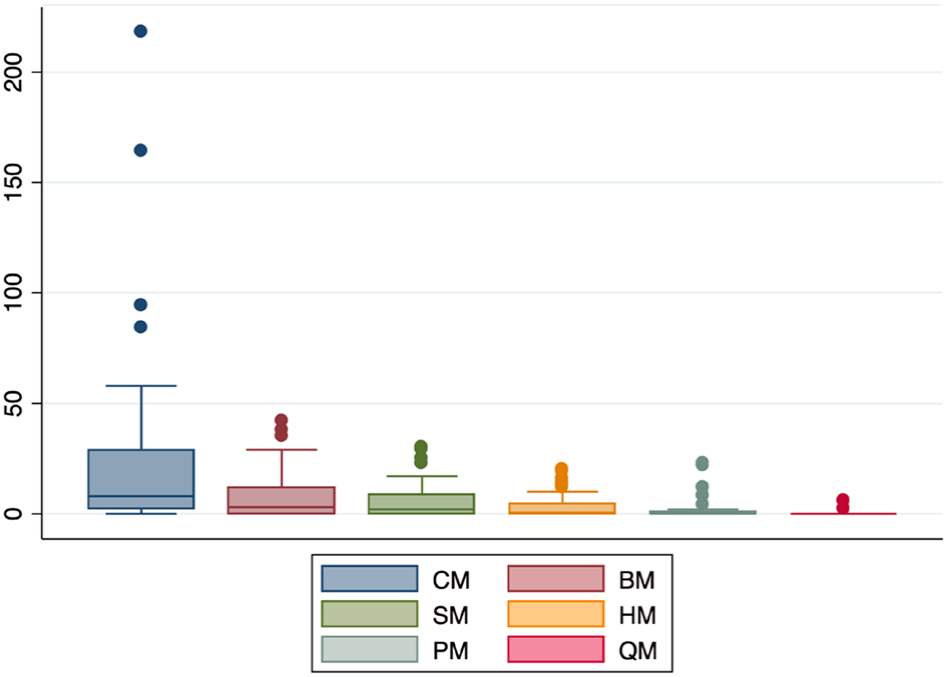
Boxplots of number of manure-inhabiting mites by manure type: cattle (CM), buffalo (BM), sheep (SM), horse (HM), poultry (PM) and quail (QM). Median values as central lines, box range with upper and lower quartiles and outsiders as dots

In total, 1892 mites belonging to 40 species, 24 genera and 16 families were sampled from the five domestic animal manure types. The highest number of mites was found in manure of cattle, followed by buffalo, sheep, horse, poultry and finally quail, with just a handful (Fig. 1).

**Fig. 2:**
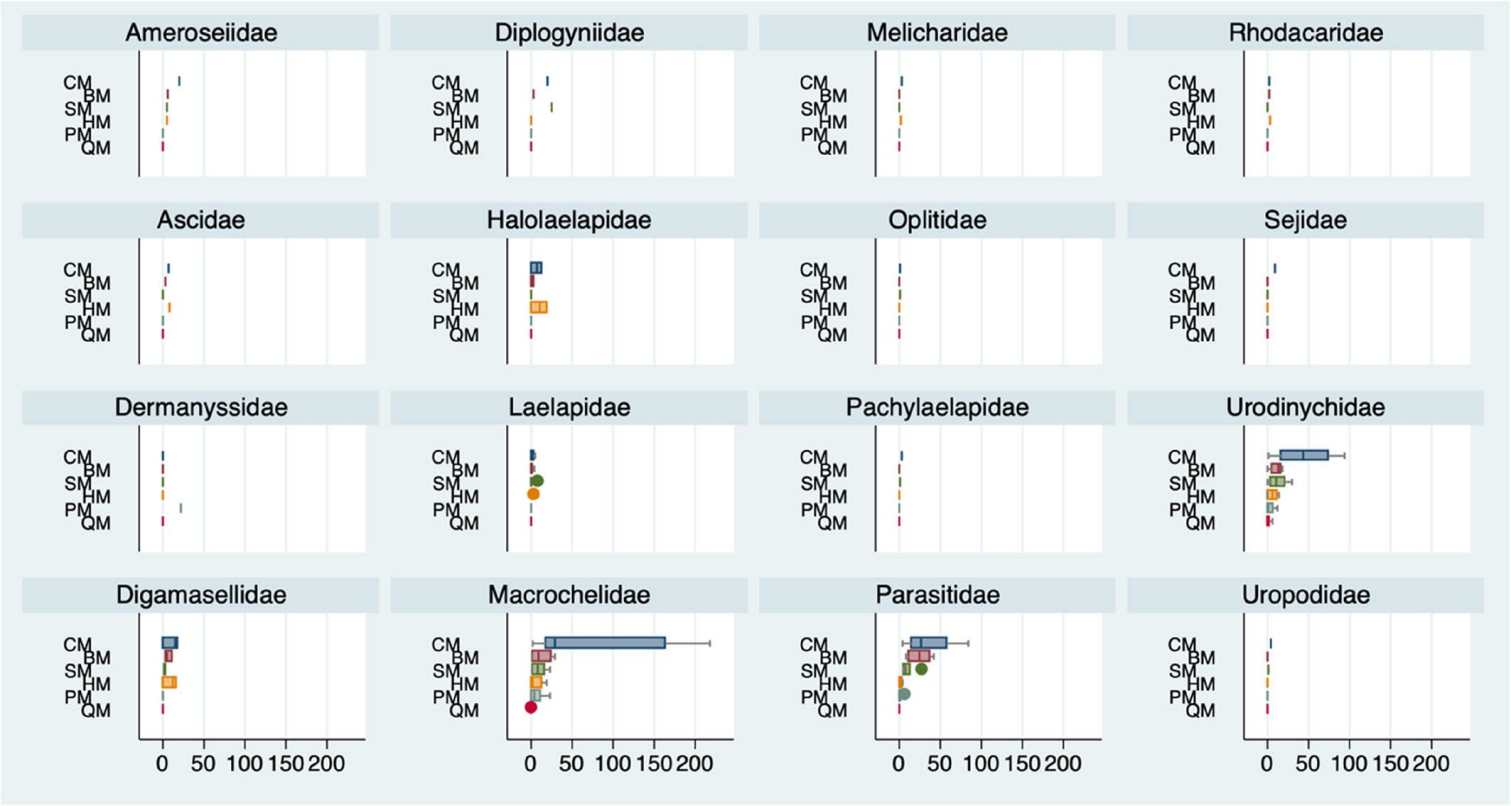
Boxplots of number of mites per manure type by Mesostigmata family: cattle (CM), buffalo (BM), sheep (SM), horse (HM), poultry (PM) and quail (QM). Median values as central lines, box range includes upper and lower quartiles and outsiders as dots

Overall, Macrochelidae was the predominant family (abundance) followed by Urodinychidae and Parasitidae (Fig. 2). In terms of species diversity, Laelapidae with up to eight species, Macrochelidae with seven and Parasitidae with six contained the highest species diversity among the 16 recorded families.

According to relative frequency of species found (Table 1), *M. muscaedomesticae*, *Parasitus beta*, *Uroobovella marginata*, *Halolaelaps sexclavatus*, *Macrocheles merdarius* and *U*. *marginata* were the dominant species within cattle (Table S1), buffalo (Table S2), sheep (Table S3), horse (Table S4), poultry (Table S5) and quail manure (Table S6), respectively. *Proctolaelaps ventrianalis* was recorded for the first time from Khuzestan. *Macrocheles muscaedomesticae* and *U. marginata* were the most widespread species which were recorded in 28 and 27 out of 30 collection sites, respectively. On the other hand, *Uroobovella varians* and *Gaeolaelaps minor* were collected only in one site. *Kleemannia parplumosa*, *Onchodellus karawaiewi* and *Dermanyssus gallinae* were recorded for the first time associated with manure in southwest Iran.

**Table 1:** Species of Mesostigmata mites and their relative frequency (%) in each of six manure types

Species	Family	Manure types					
		Cattle	Buffalo	Sheep	Horse	Poultry	Quail
*Kleemannia parplumosa* Nasr & Abou-Awad	Ameroseiidae	1.85	1.83	2.10	3.47		
Ascidae sp.	Ascidae	0.64	0.91		5.55		
*Dermanyssus gallinae* (De Geer)	Dermanyssidae					**23.15**	
*Dendrolaelaps acriluteus* Athias-Henriot	Digamasellidae	1.38	1.52	0.84	8.33		
*Dendrolaelaps multidentatus* (Leitner)	Digamasellidae	1.66	3.66	0.84	11.11		
*Dendrolaelaps presepum* Berlese	Digamasellidae		0.91	1.26			
*Lobogynium sudhiri* (Datta)	Diplogyniidae	1.85	0.91	**10.50**			
*Halolaelaps sexclavatus* (Oudemans)	Halolaelapidae	1.29	1.22		**13.88**		
*Leitneria pugio* (Karg)	Halolaelapidae				0.69		
*Androlaelaps casalis* (Berlese)	Laelapidae		0.30	0.84			
*Androlaelaps projecta* Furman	Laelapidae		0.61				
*Androlaelaps shealsi* Costa	Laelapidae		1.22				
*Androlaelaps* sp.	Laelapidae	0.27					
*Cosmolaelaps brevipedestra* (Karg)	Laelapidae	0.46					
*Gaeolaelaps khajooii* Kazemi, Rajaei & Beaulieu	Laelapidae	0.18					
*Gaeolaelaps minor* Costa	Laelapidae	0.18					
*Hypoaspisella linteyini* Samšiňák	Laelapidae	0.27	0.61	3.78	3.47		
*Glyptholaspis confuse* (Foà)	Macrochelidae	8.70	4.58	5.04		8.42	
*Macrocheles glaber* (Müller)	Macrochelidae	2.59	2.75	0.42	1.38	4.21	
*Macrocheles merdarius* (Berlese)	Macrochelidae	**15.18**	8.86	9.66	**13.19**	**24.21**	
*Macrocheles muscaedomesticae* (Scopoli)	Macrochelidae	**20.18**	7.33	7.14	9.02	2.10	
*Macrocheles scutatus* (Berlese)	Macrochelidae	1.66	2.14	1.68			
*Macrocheles subbadius* (Berlese)	Macrochelidae	0.18	0.30	0.84			
*Proctolaelaps ventrianalis* Karg	Melicharidae	0.27			1.38		
*Oplitis paradoxa* (Ganestrini & Berlese)	Oplitidae	0.09		0.42			
*Onchodellus karawaiewi* (Berlese)	Pachylaelapidae	0.27		0.42			
*Cornigamasus ocliferius* Skorupski et Witaliński	Parasitidae	5.37	**10.70**	2.52		2.10	
*Parasitus beta* Oudemans & Voigts	Parasitidae	3.24	**12.84**	3.78	2.08		
*Parasitus fimetorum* (Berlese)	Parasitidae	7.77	**11.62**	**12.18**	0.69	8.42	
*Parasitus* sp.	Parasitidae	1.38	2.44	2.52	2.77		
*Rhabdocarpais mammillatus* (Berlese)	Parasitidae	1.66	4.28	5.88			
*Trachygamasus karuna* Farahi & Witaliński	Parasitidae	0.37	3.66	2.10	0.69		
Rhodacaridae sp.	Rhodacaridae	0.18	0.61		2.08		
*Sejus australis* Hirschmann & Kaczmarek	Sejidae	0.83					
*Uroobovella difoveolata* Hirschmann & Zirngiebl-Nicol	Urodinychidae	5.09	3.05	5.4621	1.38		
*Uroobovella fimicola* (Berlese)	Urodinychidae	2.96	5.50	3.3613	6.94	2.10	
*Uroobovella marginata* (CL Koch)	Urodinychidae	8.70	4.89	**12.6050**	9.72	**12.63**	**75**
*Uroobovella varians* Hirschmann & Z.-Nicol	Urodinychidae	0.09					
*Uropoda orbicularis* (Müller)	Uropodidae	0.37		0.4201			

The number of shared species between two manure types ranged from a maximum of 24 species, such as for cattle and sheep or cattle and buffalo, to a minimum of two species, for quail and all other manure types. Interestingly, only two species, *Macrocheles sumbaensis* and *U. marginata*, were found in all studied manure types.

**Fig. 3:**
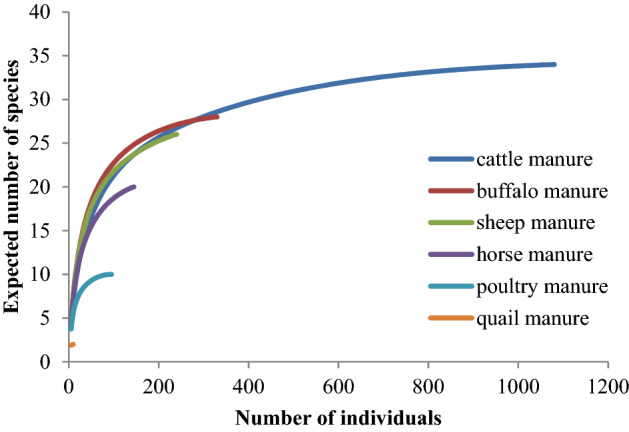
Theoretical manure-inhabiting mite species richness curves based on rarefaction method, comparing samples taken from six manure types of studied regions in Khuzestan province, southwest Iran

The rarefaction curves of manure-inhabiting mesostigmatid mites in southwestern Iran estimated the number of expected species for the 95 collection sites of the studied manure types as 22.47 species for buffalo, 21.51 species for sheep, 20.95 species for cattle, 18.50 species for horse and 10 species for poultry manure (Fig. 3). Species richness of cattle dung would be higher than buffalo when number of individuals goes beyond 300. The lowest richness was observed in quail manure.

The highest species richness was recorded in cattle (34 species) and buffalo (28 species) manure, followed by sheep, horse, poultry and quail (Table 2). However, the mite community in buffalo and sheep manure were significantly more diverse than cattle manure, according to Simpson’s diversity index. Quail manure was found to be the most uninhabited, the least diverse in our results. Simpson’s evenness in quail and poultry were higher than other manure types. Cattle manure showed the lowest evenness value (Table 2).

**Table 2:** Sample size, number of species, Simpson’s diversity index and Simpson’s evenness index for the manure-inhabiting Mesostigmata mites within six manure types in Ahvaz and its suburbs

Manure type	No. samples	No. species	Simpson’s diversity	Simpson’s evenness
Cattle	33	34	0.904 b	0.306
Buffalo	27	28	0.929 a	0.504
Sheep	20	26	0.927 a	0.525
Horse	6	20	0.912 ab	0.577
Poultry	10	10	0.838 c	0.619
Quail	4	2	0.410 d	0.800

Values within a column followed by the same letter are not significantly different (randomisation test with 10,000 re-samples: P > 0.05)

The taxonomic distinctness index (Δ*) for cattle, buffalo, sheep, horse, poultry and quail manure was 3.58, 3.65, 3.75, 3.83, 3.63 and 4.00, respectively. Horse and sheep manure showed the most taxonomic diversity among manure types. Quail should be considered cautiously due to the estimations being based on four samples and two species only.

## Discussion

The great species diversity found in the families Laelapidae, Macrochelidae and Parasitidae that inhabit manure was originally discovered by Leitner (1946a), who listed 121 manure mites from Eastern Alps. Arjomandi et al. (2013) also reported similar results on diversity of the manure-inhabiting Mesostigmata in Kerman County, southeastern Iran. Our results indicated the highest abundance of mites in cattle manure, with 34 species. The humidity content of cattle manure is kept higher for longer than in sheep and poultry, and this holding capacity seems to have a positive effect on the community of coprophilous gamasid mites (Kamaruzaman et al. 2018; Perotti 2001). In addition, the ratio of carbon/nitrogen (C/N) in cattle manure (19/1) is higher than in sheep (16/1) and poultry manure (4/1) which may represent another factor affecting the abundance of mites in dung in both studies (Augustin and Rahman 2010).

According to the rarefaction analysis, with a higher sampling size the number of expected species would increase, although for quail manure mites the results are inconclusive due to lack of data. For quail manure it seems that sample size was very small, due to the size of the quail manure areas  themselves, therefore, more data will be needed to enable a comparison of quail manure mites with other manure types. Real (Table 2) and theoretical species richness (Fig. 3) were congruent especially in higher number of individuals. Low abundance of some species may be due to sampling area swifts, or size (e.g., like the case for quail manure). Taxonomic distinctness quantifies diversity as the relatedness of the species within a sample, based on the distances between species in a classification tree (Magurran 2004). Communities may be identical in terms of richness and evenness but differ in taxonomic diversity of taxa, species. Taxonomic distinctness has been used as a tool to examine ecological degradation in marine environments and sampling methods of invertebrates (Baños-Picón et al. 2009; Tolimieri and Anderson 2010). Horse and poultry manure showed the highest values of taxonomic distinctness, despite cattle, buffalo and sheep holding the highest number of mites. This might be due to the phoretic arrival onto the manure types, as different insect carriers will visit horse and poultry manure. Most phoretic mites associated to manure are carrier-species or family specific (Axtell 1963a; Krantz 1983, 1998). This study did not sample carriers of mites, therefore no assumptions can be made on the potential insect carriers of mites for the various manure types.

In our current study Parasitidae mite species are more prevalent in cattle, buffalo and sheep than in horse and poultry droppings. A study in the Philippines showed that parasitids were more abundant in caraboa (water buffalo), dairy cattle, and swine manure than in poultry droppings (De Jesus and Rueda 1992). The parasitid mite *Poecilochirus monospinosus* was reported to prey on house fly juveniles in poultry manure (Wise et al. 1988). This predator is found mostly in late spring and early summer, and was considered to be only a minor, short-term factor in suppressing fly populations (Geden et al. 1988).

Several species of the Urodinychidae, specially *U. marginata* were found abundant in all types of manure investigated. This species is a slow-moving mite that has been well studied in poultry and cattle manure (Gerson et al. 2003). It seems to be broadly adapted to the manure and soil habitats, as suggested by Anderson (1977). It survives on many live and dead organic diets, including fly larvae, nematodes and fungi (Faash 1967). Reproduction is sexual and eggs are oviposited only by fertilized females. The females had a long, 7-month preoviposition period (Jalil and Rodriguez 1970). Perotti (2001) compared the predatory strategies of macrochelids such as *M. muscaedomesticae* and uropodids, and proposed that *U. marginata* occurs in the same substrate or manure, feeding on immature fly stages; however, both differ in their prey location and preying strategies. Whereas *M. muscaedomesticae* will go after fly eggs and first instars located on the surface, *U. marginata* will group in ‘gangs’ that hunt first and second instars trying to hide into the manure. The uropodid strategy was also proposed by Willis and Axtell (1968). Other macrochelids, such as *Glyptholaspis confusa*, shelter deeper inside the manure where they prey on hidden eggs (Perotti 2001).

Macrochelidae species were also highly numerous. *Macrocheles muscaedomesticae* and *M. merdarius* were found abundantly in five of the six tested manure types (except quail manure). Similarly, it has been reported that approximately 450 species of mites representing 18 families and 48 genera in three orders are known to be associated with animal dungs in the world. Over 60% of these mites, or 280 species, are macrochelids (Krantz 1983). These mites reduce pest flies. For instance, *M. muscaedomesticae* is a well-known predator of the house fly *M. domestica*, the face fly *Musca automnalis* De Geer, the stable fly *Stomoxys calcitrans* (L.), and the horn fly *Haematobia irritants* (L.) (Axtell 1963b; De Jesus and Rueda 1992; Filipponi 1964; Geden et al. 1988; Perotti 1999b, 2000b, 2001). In this regard, Axtell (1963b) reported that *Macrocheles* species can reduce the pest flies more efficiently than any pesticides. Similarly, Rodriguez et al. (1970) showed that when machrochelid species were added to manure, they could decrease the density of the pest fly population by approximately 90%.

The only two species present in all types of manure were *M. sumbaensis* and *U. marginata*. Two of the most frequent species were *M. muscaedomesticae* and *U. marginata*; and this is in line with early studies. Anderson (1983) reported that in the USA both species are common predators of pest fly eggs, larvae, or both, in agricultural systems characterized by confined, high-density concentrations of livestock and poultry. The role and importance of *M. muscaedomesticae* and *U. marginata* in integrated pest management (IPM) programs has been already confirmed (Anderson 1983; Axtell 1968, 1970b; Geden et al. 1990; Rodriguez et al. 1970; Wicht and Rodriguez 1970). The main reason why *M. muscadomestica* and *U. marginata* currently offer the highest potential as effective predators is that they occur together in the same manure, complementing each other. Both prey on key pest fly species associated with confined livestock and poultry productions worldwide, and both species can be mass-reared for inundative or inoculative releases. Furthermore, to a large degree these species exhibit the four essential characteristics itemized by Doutt and Debach (1964) of an effective natural enemy: (1) high searching/finding ability; (2) high degree of host (prey) specificity; (3) high rate of increase in relation to the pest population; and (4) capability of dwell in all of the host- (or prey-)inhabited microhabitats. Parasitidae are almost as ubiquitous as Macrochelidae in manure, therefore they should also be included in pest control plans.

Although the mite species *K. parplumosa*, *O. karawaiewi* and *D. gallinae* were not know from manure in the studied region, they have been recorded previously from soil (Farahi et al. 2018).

This work reports the occurrence of up to seven species of Macrochelidae in the manure of livestock and poultry productions in southwest Iran (Ahvaz and suburbs), two of them in high numbers, *M. muscaedomesticae* and *M. sumbaensis*, the latter with a 100% prevalence. They co-inhabit the manure together with *U. marginata* and key Parasitidae species. The findings suggest that the most relevant predators of filthy flies are in place and adapted, bringing the possibility to use or consider them in future pest control plans. However, the biology and predatory traits of many of these species, e.g., of the abundant *M. sumbaensis*, should be studied or revised to further assess their feasibility as biocontrol agents of filth flies.

## Electronic Supplementary Material

Below is the link to the electronic supplementary material.

Supplementary Material 1

## Data Availability

All numerical data is disclosed in the Supplementary Tables file (Tables S1 to S6).
