# Supplementary material for: Mesostigmata diversity by manure type: a reference study and new datasets from southwestern Iran
Source: Exp Appl Acarol. 2022 Mar 31;86(4):517–34. doi: 10.1007/s10493-022-00710-1 (PMC9110456; doi:10.1007/s10493-022-00710-1)
Supplement: Supplementary file 1 — Supplementary Material 1 [file 10493_2022_710_MOESM1_ESM.pdf]

Mesostigmata diversity by manure type. A reference study and new datasets from southwestern Iran

Sara Farahi, Parviz Shishehbor, Alireza Nemati, Alejandra Perotti

Corresponding authors: Sara Farahi sara.farahi@gmail.com; Department of Plant Protection, Faculty of Agriculture, Shahid Chamran University of Ahvaz, Ahvaz, Iran
M. Alejandra Perotti m.a.perotti@reading.ac.uk; Ecology and Evolutionary Biology Section, School of Biological Sciences, University of Reading, United Kingdom

Table 1. List, numbers of individuals of Mesostigmata mites associated with cattle manure.

Species	Family	Number
Kleemannia parplumosa Nasr & Abou-Awad, 1986	Ameroseiidae	20
Proctolaelaps ventrianalis Karg, 1971	Melicharidae	3
Androlaelaps sp.	Laelapidae	3
Cosmolaelaps brevipedestra (Karg, 1985)	Laelapidae	5
Gaeolaelaps khajooii Kazemi, Rajaei & Beaulieu, 2014	Laelapidae	2
Gaeolaelaps minor Costa, 1968	Laelapidae	2
Hypoaspisella linteyini Samšiňák, 1964	Laelapidae	3
Glyptholaspis confusa (Foà, 1900)	Macrochelidae	94
Macrocheles glaber (Müller, 1860)	Macrochelidae	28
Macrocheles merdarius (Berlese, 1889)	Macrochelidae	164
Macrocheles muscaedomesticae (Scopoli, 1772)	Macrochelidae	218
Macrocheles scutatus (Berlese, 1904)	Macrochelidae	18
Macrocheles subbadius (Berlese, 1904)	Macrochelidae	2
Macrocheles sumbaensis Hartini & Takaku, 2005	Macrochelidae	29
Onchodellus karawaiewi (Berlese, 1920)	Pachylaelapidae	3
Halolaelaps sexclavatus (Oudemans, 1902)	Halolaelapidae	14
Dendrolaelaps acriluteus Athias-Henriot, 1961	Digamasellidae	15
Dendrolaelaps multidentatus (Leitner, 1949)	Digamasellidae	18
Trachygamasus karuni Farahi & Witaliński, 2019	Parasitidae	4
Cornigamasus ocliferius Skorupski et Witaliński, 1997	Parasitidae	58
Rhabdocarpais mammillatus (Berlese, 1904)	Parasitidae	18
Parasitus beta Oudemans & Voigts, 1904	Parasitidae	35
Parasitus fimetorum (Berlese, 1904)	Parasitidae	84
Parasitus sp.	Parasitidae	15
Oplitis paradoxa (Ganestrini & Berlese, 1884)	Oplitidae	1
Uroobovella difoveolata Hirschmann & Zirngiebl-Nicol, 1962	Urodinychidae	55
Uroobovella fimicola (Berlese, 1903)	Urodinychidae	32
Uroobovella marginata (C.L.Koch, 1839)	Urodinychidae	94
Uroobovella varians Hirschmann & Z.-Nicol, 1962	Urodinychidae	1
Uropoda orbicularis (Müller, 1776)	Uropodidae	4
Lobogynium sudhiri (Datta,1985)	Diplogyniidae	20
Sejus australis Hirschmann & Kaczmarek, 1991	Sejidae	9
Ascidae sp.	Ascidae	7
Rhodacaridae sp.	Rhodacaridae	2

Table 2. List, numbers of individuals of Mesostigmata mites associated with buffalo manure.

Species	Family	Number
Kleemannia parplumosa Nasr & Abou-Awad, 1986	Ameroseiidae	6
Androlaelaps casalis (Berlese, 1887)	Laelapidae	1
Androlaelaps projecta Furman, 1972	Laelapidae	2
Androlaelaps shealsi Costa, 1968	Laelapidae	4
Hypoaspisella linteyini Samšiňák, 1964	Laelapidae	2
Glyptholaspis confusa (Foà, 1900)	Macrochelidae	15
Macrocheles glaber (Müller, 1860)	Macrochelidae	9
Macrocheles merdarius (Berlese, 1889)	Macrochelidae	29
Macrocheles muscaedomesticae (Scopoli, 1772)	Macrochelidae	24
Macrocheles scutatus (Berlese, 1904)	Macrochelidae	7
Macrocheles subbadius (Berlese, 1904)	Macrochelidae	1
Macrocheles sumbaensis Hartini & Takaku, 2005	Macrochelidae	2
Halolaelaps sexclavatus (Oudemans, 1902)	Halolaelapidae	4
Dendrolaelaps acriluteus Athias-Henriot, 1961	Digamasellidae	5
Dendrolaelaps multidentatus (Leitner, 1949)	Digamasellidae	12
Dendrolaelaps presepum Berlese, 1918	Digamasellidae	3
Trachygamasus karuni Farahi & Witaliński, 2019	Parasitidae	12
Cornigamasus ocliferius Skorupski et Witaliński, 1997	Parasitidae	35
Rhabdocarpais mammillatus (Berlese, 1904)	Parasitidae	14
Parasitus beta Oudemans & Voigts, 1904	Parasitidae	42
Parasitus fimetorum (Berlese, 1904)	Parasitidae	38
Parasitus sp.	Parasitidae	8
Uroobovella difoveolata Hirschmann & Zirngiebl-Nicol, 1962	Urodinychidae	10
Uroobovella fimicola (Berlese, 1903)	Urodinychidae	18
Uroobovella marginata (C.L.Koch, 1839)	Urodinychidae	16
Lobogynium sudhiri (Datta,1985)	Diplogyniidae	3
Ascidae sp.	Ascidae	3
Rhodacaridae sp.	Rhodacaridae	2

Table 3. List, numbers of individuals of Mesostigmata mites associated with sheep manure.

Species	Family	Number
Kleemannia parplumosa Nasr & Abou-Awad, 1986	Ameroseiidae	5
Androlaelaps casalis (Berlese, 1887)	Laelapidae	2
Hypoaspisella linteyini Samšiňák, 1964	Laelapidae	9
Glyptholaspis confusa (Foà, 1900)	Macrochelidae	12
Macrocheles glaber (Müller, 1860)	Macrochelidae	1
Macrocheles merdarius (Berlese, 1889)	Macrochelidae	23
Macrocheles muscaedomesticae (Scopoli, 1772)	Macrochelidae	17
Macrocheles scutatus (Berlese, 1904)	Macrochelidae	4
Macrocheles subbadius (Berlese, 1904)	Macrochelidae	2
Macrocheles sumbaensis Hartini & Takaku, 2005	Macrochelidae	8
Onchodellus karawaiewi (Berlese, 1920)	Pachylaelapidae	1
Dendrolaelaps acriluteus Athias-Henriot, 1961	Digamasellidae	2
Dendrolaelaps multidentatus (Leitner, 1949)	Digamasellidae	2
Dendrolaelaps presepum Berlese, 1918	Digamasellidae	3
Trachygamasus karuni Farahi & Witaliński, 2019	Parasitidae	5
Cornigamasus ocliferius Skorupski et Witaliński, 1997	Parasitidae	6
Rhabdocarpais mammillatus (Berlese, 1904)	Parasitidae	14
Parasitus beta Oudemans & Voigts, 1904	Parasitidae	9
Parasitus fimetorum (Berlese, 1904)	Parasitidae	29
Parasitus sp.	Parasitidae	6
Oplitis paradoxa (Ganestrini & Berlese, 1884)	Oplitidae	1
Uroobovella difoveolata Hirschmann & Zirngiebl-Nicol, 1962	Urodinychidae	13
Uroobovella fimicola (Berlese, 1903)	Urodinychidae	8
Uroobovella marginata (C.L.Koch, 1839)	Urodinychidae	30
Uropoda orbicularis (Müller, 1776)	Uropodidae	1
Lobogynium sudhiri (Datta,1985)	Diplogyniidae	25

Table 4. List, numbers of individuals of Mesostigmata mites associated with horse manure.

Species	Family	Number
Kleemannia parplumosa Nasr & Abou-Awad, 1986	Ameroseiidae	5
Proctolaelaps ventrianalis Karg, 1971	Melicharidae	2
Hypoaspisella linteyini Samšiňák, 1964	Laelapidae	5
Macrocheles glaber (Müller, 1860)	Macrochelidae	2
Macrocheles merdarius (Berlese, 1889)	Macrochelidae	19
Macrocheles muscaedomesticae (Scopoli, 1772)	Macrochelidae	13
Macrocheles sumbaensis Hartini & Takaku, 2005	Macrochelidae	3
Halolaelaps sexclavatus (Oudemans, 1902)	Halolaelapidae	20
Leitneria pugio (Karg, 1961)	Halolaelapidae	1
Dendrolaelaps acriluteus Athias-Henriot, 1961	Digamasellidae	12
Dendrolaelaps multidentatus (Leitner, 1949)	Digamasellidae	16
Trachygamasus karuni Farahi & Witaliński, 2019	Parasitidae	1
Parasitus beta Oudemans & Voigts, 1904	Parasitidae	3
Parasitus fimetorum (Berlese, 1904)	Parasitidae	1
Parasitus sp.	Parasitidae	4
Uroobovella difoveolata Hirschmann & Zirngiebl-Nicol, 1962	Urodinychidae	2
Uroobovella fimicola (Berlese, 1903)	Urodinychidae	10
Uroobovella marginata (C.L.Koch, 1839)	Urodinychidae	14
Ascidae sp.	Ascidae	8
Rhodacaridae sp.	Rhodacaridae	3

Table 5. List, numbers of individuals of Mesostigmata mites associated with poultry manure.

Species	Family	Number
Dermanyssus gallinae (De Geer, 1778)	Dermanyssidae	22
Glyptholaspis confusa (Foà, 1900)	Macrochelidae	8
Macrocheles glaber (Müller, 1860)	Macrochelidae	4
Macrocheles merdarius (Berlese, 1889)	Macrochelidae	23
Macrocheles muscaedomesticae (Scopoli, 1772)	Macrochelidae	2
Macrocheles sumbaensis Hartini & Takaku, 2005	Macrochelidae	12
Cornigamasus ocliferius Skorupski et Witaliński, 1997	Parasitidae	2
Parasitus fimetorum (Berlese, 1904)	Parasitidae	8
Uroobovella fimicola (Berlese, 1903)	Urodinychidae	2
Uroobovella marginata (C.L.Koch, 1839)	Urodinychidae	12

Table 6. List, numbers of individuals of Mesostigmata mites associated with quail manure.

Species	Family	Number
Macrocheles sumbaensis Hartini & Takaku, 2005	Macrochelidae	2
Uroobovella marginata (C.L.Koch, 1839)	Urodinychidae	6
